# Mechanisms of Viral DNA Replication of Human Papillomavirus: E2 Protein-Dependent Recruitment of E1 DNA Helicase to the Origin of DNA Replication

**DOI:** 10.3390/ijms26094333

**Published:** 2025-05-02

**Authors:** Anshul Rana, Gulden Yilmaz, Esther E. Biswas-Fiss, Subhasis Biswas

**Affiliations:** 1 Department of Medical and Molecular Sciences, College of Health Sciences, University of Delaware, Newark, DE 19716, USA; rana@udel.edu (A.R.); ebiswas@udel.edu (E.E.B.-F.); 2 Ammon Pinizzotto Biopharmaceutical Innovation Center, Newark, DE 19713, USA; 3 Department of Molecular Biology, Rowan University, Stratford, NJ 08084, USA

**Keywords:** HPV, DNA replication, DNA helicase, E1 helicase, E2 protein, LCR, pre-initiation complex, replisome formation, papillomavirus mechanism

## Abstract

Human papillomaviruses (HPVs) are small double-stranded DNA viruses that infect epithelial cells and cause cervical, anogenital, and oropharyngeal cancers. HPV genome replication relies on the viral E1 and E2 proteins to initiate DNA replication. The first step is the assembly of the E1-E2 complex at the origin of replication. We have examined the role of full-length HPV E1 helicase and its interaction with E2 in pre-initiation complex formation. Electrophoretic mobility shift assays (EMSAs) with purified E1 and E2 proteins revealed that the HPV genome does not have a specific E1 binding site, or such a sequence is not required for pre-initiation complex formation. E1 alone did not show any binding to the origin DNA sequences, while E2 facilitated E1 recruitment to the origin, forming the E1-E2-DNA ternary complex. Formation of such a complex required at least two E2 binding sites. These findings led us to propose a novel mechanism in which E2 dimers serve as the primary recruiters of E1 to form the pre-initiation complex. This study provides new insights into the mechanistic role of E2 in the recruitment of E1 at the origin of HPV DNA replication, enhancing our understanding of HPV biology and potentially informing future therapeutic strategies.

## 1. Introduction

Human papillomavirus (HPV) is a family of small, circular, double-stranded DNA viruses that infect and proliferate in the epithelial cells. It is likely the most ancient (>400 million years) virus with nearly 215 known subtypes, with presence in every continent. HPVs are the most common viral agents responsible for causing a wide range of cancers, including cervical, anal, oropharyngeal, and other genital, head, and neck malignancies [1,2,3,4,5,6]. The alpha papillomavirus genus, with 65 distinct HPV types, in particular, includes both low-risk and high-risk subtypes that vary in their carcinogenic potential in humans [7]. Low-risk HPV types, such as HPV6 and HPV11, are associated with benign conditions, including low-grade cervical abnormalities and anogenital warts. In contrast, high-risk subtypes, such as HPV16 and HPV18, are responsible for the majority of cervical cancers, accounting for approximately 70% of cervical cancer cases globally and a large proportion of oropharyngeal carcinomas [8,9,10].

The HPV genome codes for several viral genes, divided into early and late regions based on their temporal expression during the viral life cycle [11,12]. Upon infection of basal epithelial cells, the viral genome is established as multicopy episomes, maintaining between 50 and 100 copies per host cell [13]. The episomal replication of the viral DNA requires two virus-encoded proteins, E1 and E2, along with host replication factors, such as DNA polymerases alpha and epsilon for DNA synthesis, replication protein A for single-stranded DNA binding, and topoisomerase I to relax the supercoiled DNA during viral DNA replication [14,15,16,17,18,19]. The HPV genome also includes a long control region (LCR), which varies in size among papillomaviruses, with lengths of 853 bp in HPV16, 825 bp in HPV18, and 940 bp in BPV1 [20]. The LCR contains binding sites for the E2 initiator protein and an AT-rich sequence that serves as the origin of replication [21]. The origin includes four E2 binding sites with the consensus sequence 5′-ACCG(N)_4_CGGT-3′. High-risk HPVs often exhibit single-nucleotide variations (SNVs) in binding site 3 (BS3), particularly in the CGGT sequence, leading to reduced E2 binding affinity, impaired origin activation, and replication. In contrast, low-risk HPVs maintain conserved binding sequences, enabling efficient replication. These sequence differences in origin regions are associated with the differential oncogenic potential of HPV strains [22].

E2 is a multifunctional viral protein that plays a critical role in viral genome maintenance, gene expression, and DNA replication [21,23,24]. Within the LCR, the E2 protein binds to four binding sites, each containing the consensus sequence 5′-ACCG(N)_4_CGGT-3′ [22]. E2 binds as a dimer at each of these binding sites with varying affinities, ranging from 4.8 to 10.2 nM dissociation constants for the different sites [25]. E2 also functions as a transcriptional regulator, binding to specific DNA sequences within the LCR, including the promoters p97 and p105, which control the expression of oncogenes E6 and E7 [21].

E1 is the DNA helicase for HPV DNA replication [26,27,28,29,30]. As a member of the superfamily III (SF3) of helicases, E1 comprises five domains: the amino-terminal, DNA-binding, ATPase, helicase, and carboxy-terminal domains [31]. E1 is encoded by the largest and most conserved open-reading frame (ORF) of the papillomavirus genome, with the protein ranging from 600 to 650 amino acids depending on the HPV type. The N-terminal region, comprising approximately 200 amino acids, is the least conserved segment of E1 and includes variably conserved motifs such as a nuclear localization signal, a nuclear export signal, and a cyclin-binding motif, which vary across different HPV types [31,32]. The oligomerization domain is highly conserved, with four highly conserved regions among papillomaviruses, which are critical for E1 oligomerization in vitro and supporting transient DNA replication in vivo [33]. The ATPase domain, which is the most conserved segment of the E1 protein, contains the Walker A and Walker B motifs, as well as a conserved arginine finger, which likely plays a critical role in coupling ATP hydrolysis to the conformational changes required for DNA unwinding [32,34,35]. The helicase and DNA-binding domains are crucial for the interaction of E1 with essential host factors, including DNA polymerase alpha, topoisomerase I, and replication protein A (RPA). Upon binding to the origin, E1, as a replicative DNA helicase, unwinds DNA and facilitates DNA replication [36,37,38].

The initiation of HPV DNA replication requires the assembly of a complex involving the viral E1 and E2 proteins at the origin of replication [11,12,39]. Previous studies suggest that the formation of the E1-E2 complex at the origin is site-specific, occurring when both E1 and E2 bind to their respective sites within the origin [14,40,41,42,43,44]. In contrast, the role of the E1 binding site in HPV replication remains poorly understood. Earlier studies proposed that in HPV11 and HPV31b, the E1 binding site, located between E2 binding sites 2 and 3, consists of an 18-nucleotide AT-rich palindrome, and efficient E1 binding to this site was thought to be critical for the formation of the E1-E2-origin complex [45,46,47]. E1 is a DNA helicase like the DnaB helicase of *E. coli*. In *E. coli,* the DnaA protein binds to specific sites on the origin DNA, and the DnaB helicase is loaded onto the DnaA-DNA complex. Thus, there is no essential need for E1 helicase to have a specific site(s) for DNA binding.

In this study, we report successful expression and purification of the full-length HPV E1 protein to near homogeneity. Earlier, we reported the purification of full-length E2 protein [22]. In the present study, we present a comprehensive biochemical characterization of full-length HPV E1, including ATPase and helicase activities. We demonstrated the stimulation of E1 ATPase activity and kinetic parameters by various DNA substrates. Using the full-length E1 and E2 proteins, we examined the mechanism of E1 loading to the origin of DNA replication and the essential role of the E2 protein in this process.

## 2. Results

### 2.1. Purification of Full-Length HPV E1 and E2 Proteins

**Figure 1:**
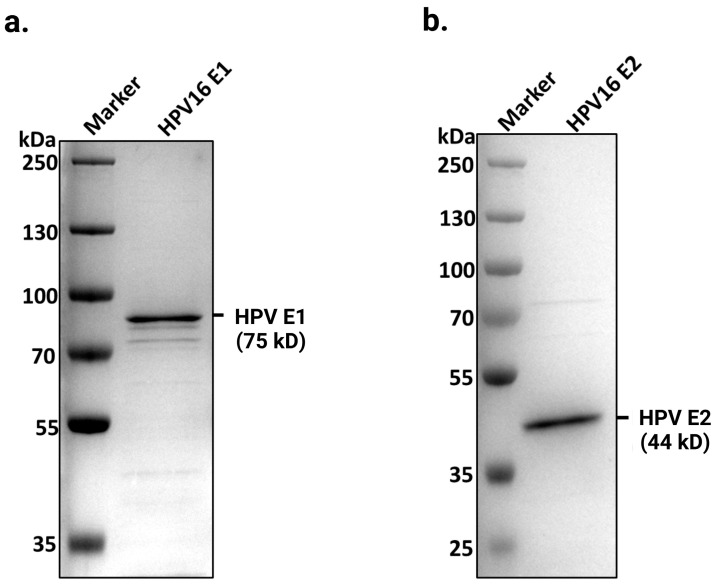
Purified full-length HPV16 E1 and E2 proteins. Coomassie blue-stained SDS-PAGE shows purified HPV16 E1 and E2 proteins at their predicted sizes of (**a**) 75 kDa and (**b**) 44 kDa, respectively, on a 4–20% gradient polyacrylamide gel. A total of 1.6 µg of E1 and 2 µg of E2 were loaded onto the gels. Proteins were purified as described in the experimental procedures.

We have purified full-length HPV E1 helicase and E2 protein following overexpression in BL21 (DE3) RIPL cells (Agilent Technologies, Santa Clara, CA, USA). Both proteins were found to be soluble only in high-salt buffer (≥500 mM NaCl), as described previously by Yilmaz et al. [22,25]. Soluble protein extracts were subjected to immobilized metal affinity chromatography to purify E1 and E2. The purified proteins were subsequently concentrated by dialysis. SDS-PAGE analysis, followed by Coomassie staining, confirmed the near-homogeneous preparations of E1 and E2, with molecular weights of approximately 75 kDa and 44 kDa, respectively (Figure 1).

### 2.2. Enzymatic Activities of Full-Length HPV E1 Helicase

#### 2.2.1. ATPase Activity of Purified E1 Helicase

**Figure 2:**
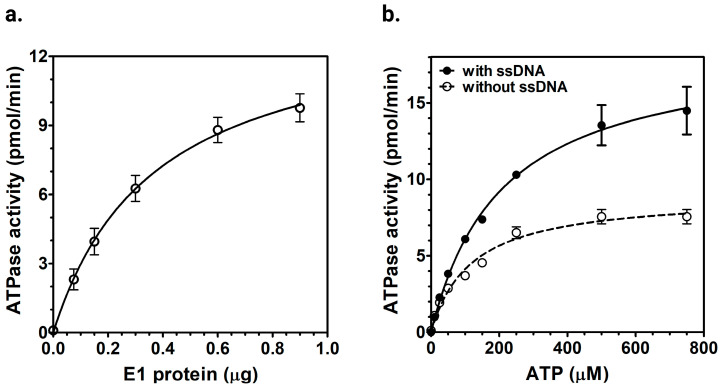
ATPase activity of E1 helicase. (**a**) ATP hydrolysis activity of E1 was measured using a standard ATPase assay with 100 μM ATP-α-^32^P and E1 protein titrations, from 0.1 to 0.9 µg. ATP hydrolysis rates are presented as pmol/min. The error bars represent the standard deviation of triplicate assays. (**b**) Kinetics of E1 ATPase activity. ATPase activity was assessed using 0.24 µg of E1, with and without 100 pmol M13mp19 ssDNA, under varying ATP concentrations (0–0.75 mM). ATP hydrolysis rates were calculated as pmol/min and analyzed using non-linear regression in Prism 5.0 to determine the kinetic parameters.

We examined the ATPase activity of the purified HPV E1 protein by measuring its activity using radiolabeled α-^32^P-ATP hydrolysis (Figure 2a). The highest ATPase activity observed in our assay was 30 nmol/min/mg. We further analyzed the kinetic parameters of E1 ATPase activity by performing non-linear regression analysis with varying ATP concentrations to calculate the *K_m_* and *V_max_* for ATPase, both in the presence and absence of M13mp19 ssDNA. In the absence of ssDNA, E1 showed a *K_m_* of 118 ± 19 μM and a *V_max_* of 37 ± 0.4 nmol/min/mg. In the presence of ssDNA, the *K_m_* and *V_max_* values shifted to 208 ± 13 μM and 78 ± 0.4 nmol/min/mg, respectively (Figure 2b). These results confirmed the DNA-dependent ATPase activity of E1 helicase.

#### 2.2.2. ssDNA-Dependent ATPase Activity of E1 Helicase

**Figure 3:**
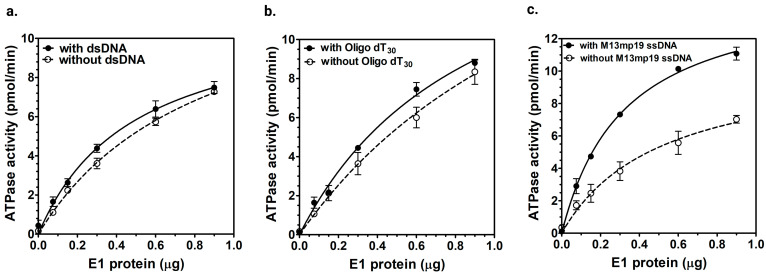
ssDNA-dependent ATPase activity of E1 helicase. ATPase activity of E1 was assessed using standard ATPase assay, as described in the experimental procedures, with 100 μM ATP-α-32P and titrations of E1 protein, as indicated, in the presence or absence of DNA cofactors. (**a**) Effect of 40 bp long dsDNA. Reactions included 100 pmol of 40 bp dsDNA to evaluate its effect on ATPase activity. (**b**) Effect of oligo dT_30_ ssDNA. Assays were performed with 100 pmol oligo dT_30_ ssDNA to determine its effect on ATPase activity of E1. (**c**) Effect of M13mp19 ssDNA. ATPase activity was measured with 100 pmol M13mp19 ssDNA as a cofactor. The ATPase activity was analyzed using non-linear regression in Prism 5.0. The error bars represent the standard deviation of triplicate assays.

To understand how ssDNA binding stimulates the ATPase activity of E1 helicase, we measured its ATPase activity with different DNA substrates: a single-stranded oligo dT_30_ DNA, a 40 bp double-stranded DNA, and a 7.25 kb long circular M13mp19 ssDNA. No significant stimulation was observed with either dsDNA or oligo dT_30_ ssDNA (Figure 3a,b). In contrast, higher ATPase activity was detected only with the M13mp19 ssDNA (Figure 3c). The increase in ATPase activity observed with M13mp19 ssDNA likely reflected that E1’s ATPase might be coupled to translocation along longer ssDNA, thereby stimulating the ATPase activity. The minimal response seen with both shorter ssDNA (oligo dT_30_) and dsDNA further highlighted possible ATP-dependent translocation on longer ssDNA.

Statistical analysis confirmed that only the M13mp19 ssDNA showed a significant increase in ATPase activity (*p* value = 0.0005), whereas dsDNA and oligo(dT)_30_ did not show significant changes (*p* = 0.7070 and 0.1947, respectively; Figure S2).

#### 2.2.3. DNA Helicase Activity of E1

**Figure 4:**
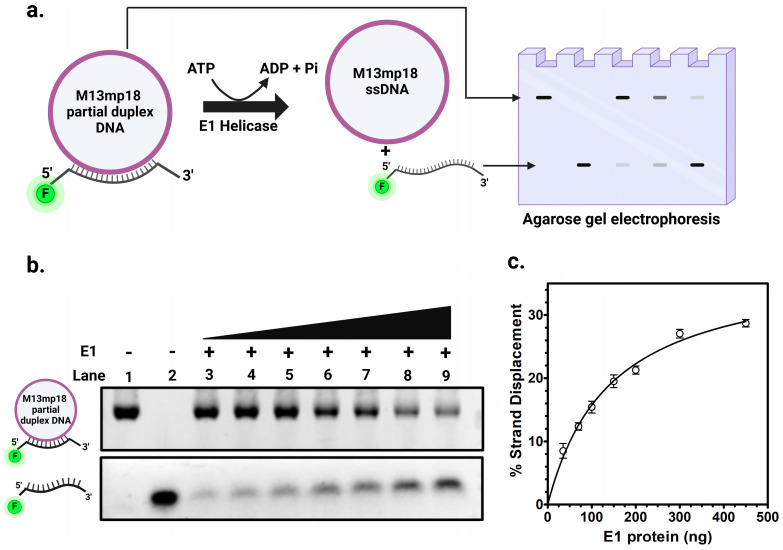
Characterization of the DNA helicase activity of E1. (**a**) Illustration of the fluorescent-based helicase assay. A 50 bp complementary oligonucleotide (labeled with 5′ FAM fluorescent tag) was annealed to M13mp18 ssDNA to create a partial duplex DNA helicase substrate. Incubation with purified E1 helicase and ATP resulted in unwinding of the ssDNA and release of the labeled oligonucleotide. The unwound products were analyzed by 1% agarose gel electrophoresis. (**b**) A representative gel from a helicase assay is shown, using a FAM-labeled partial duplex DNA substrate. Lanes 1 and 2 are controls with native and boiled partial duplex DNA substrates, respectively. Lanes 3–9 contained E1 protein titrations (44 nM, 87 nM, 125 nM, 187 nM, 250 nM, 375 nM, and 562 nM). Unwinding reactions were performed at 37 °C for 2 h, and the resulting products were separated on a 1% agarose gel in running buffer containing 1× TBE and 0.1% SDS. The analysis involved exposure of the top and bottom bands for different time periods using fluorescent channels on an iBright FL1500 Imaging System (Thermo Fisher Scientific, Waltham, MA, USA) with quantification of substrate unwinding relative to the control in lane 2, using iBright analysis software 4.0 (Thermo Fisher Scientific, Waltham, MA, USA). (**c**) Quantification of % strand displacement. Bands were quantitated as described above and plotted against corresponding E1 enzyme amounts. E1 exhibited ~28% strand displacement activity with 450 ng of purified enzyme, as determined by fluorescent band analysis.

We examined the DNA helicase activity of HPV E1 using a fluorescently labeled partial duplex DNA substrate. As illustrated in Figure 4b, the gel electrophoresis showed an increase in the unwound single-stranded DNA with increasing amounts of E1 helicase. The E1 helicase unwound a 50 bp duplex DNA, and the amount of unwinding increased proportionally with the amount of protein (Figure 4b, Lanes 3–9). The quantitative analysis of strand displacement activity of E1 helicase in Figure 4c showed that up to 28% unwinding of the input substrate was observed with E1 titration at 37 °C. These results confirmed that the purified HPV E1 exhibited functional helicase activity.

### 2.3. Mechanism of E1 Helicase Loading to the HPV Origin of DNA Replication

**Figure 5:**
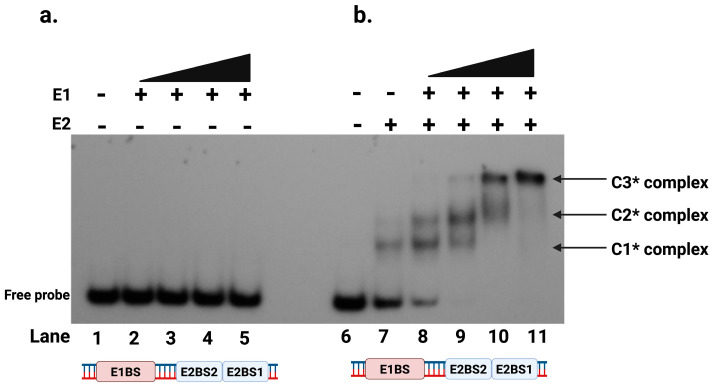
E1 binds DNA in the presence of E2. Electrophoretic mobility shift assays (EMSAs) were performed using E1, E2, and a double-stranded DNA ^32^P-labeled probe (70 pmol) containing the E1 binding site and two E2 binding sites, BS1 and BS2. The reaction mixture, including E1, E2, and ^32^P-labeled DNA probe, was incubated for 10 min at 30 °C, followed by running it on 5% native polyacrylamide gel. (**a**) The assay included a titration of E1 protein alone or (**b**) E1 titration was performed in the presence of a fixed concentration of E2 protein (2.8 nM) with 70 pmol of radiolabeled DNA probe. Increasing concentrations of E1 protein (43 nM, 87 nM, 173 nM, and 347 nM) were used. C1* represents the complex with two E2 dimers bound to DNA, C2* represents the E1-E2-DNA complex, and C3* denotes the complex containing double-hexameric E1 protein bound to the E2-DNA complex.

The HPV viral genome has a single origin of DNA replication. It is located at the LCR of the genome. The most distinguishing feature of this region is the presence of at least four 12 bp DNA binding sites for E2 protein [21]. However, the mechanism of E1 helicase loading to the origin to form a replisome remains unknown. Previous studies, primarily based on the BPV replication model, have suggested that a putative E1 binding site is required for efficient E1 binding to the origin of replication [14,40,41,42,43,44]. To study the HPV E1 helicase binding at the origin, we conducted EMSA using oligo I, a 124 bp radiolabeled DNA probe, described in Supplementary Table S1. This probe contained the putative E1 binding site and two E2 binding sites (BS1 and BS2) as present in the LCR of HPV [25]. As shown in Figure 5a, we demonstrated that E1 helicase did not bind to this DNA sequence. Even at high concentrations of E1 protein, we did not detect any binding, indicating that E1 cannot bind to the HPV origin sequence independently.

### 2.4. E2 Protein Is Required for E1 to Bind to the Protein-DNA Complex at the Origin

Next, we examined whether E1 helicase binding to the origin DNA is modulated by its cognate E2 protein. We performed EMSA using the oligo I radiolabeled DNA probe (Supplementary Table S1) containing the proposed E1 binding site and two E2 binding sites, in the presence of purified E2 protein. As previously demonstrated, E2 binds specifically to its cognate binding sites (BS1, BS2, BS3 and BS4) at the origin [21,25]. EMSA was performed with both E2 and E1 proteins to assess whether E1 could bind to the origin DNA in the presence of E2. Although E1 is not capable of binding DNA alone (Figure 5a), it appeared to bind to the E2-DNA complex (Figure 5b). In the presence of E2 protein, DNA binding was observed with E1 and E2 protein forming detectable larger protein-DNA complexes in EMSA, indicating the formation of three distinct complexes (Figure 5b). The first complex, C1*, is the E2-DNA complex, formed when E2 binds to its binding sites, with E2 dimers binding to each of the two E2 binding sites. A larger second complex, C2*, appeared on EMSA when E1 was added, indicating the cooperative binding of E1 to the E2-DNA complex. At higher E1 concentrations, an even larger third complex, C3*, was observed, representing a higher-order assembly involving double E1 hexamers binding to the E2-DNA complex (Figure 5b, Lanes 9–11).

### 2.5. E1-E2-DNA Complex Formation Did Not Require a Putative E1 Binding Site

**Figure 6:**
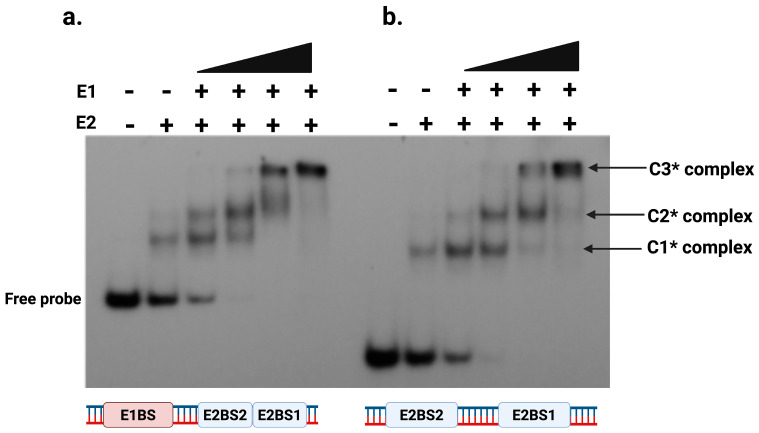
E1-E2-DNA complex formation is independent of the E1 binding site. Electrophoretic mobility shift assays (EMSAs) were performed to evaluate E1-E2-DNA complex formation using purified E1 and E2 proteins with radiolabeled dsDNA probes. (**a**) E2 BS1 + 2 + E1BS DNA Probe. The DNA probe contained two E2 binding sites (BS1 and BS2) and the E1 binding site. Reactions included a fixed ^32^P-labeled DNA probe (70 pmol), a constant E2 concentration (2.8 nM), with increasing E1 concentrations (43 nM, 87 nM, 173 nM, and 347 nM). (**b**) E2 BS1 + 2 DNA probe. The DNA probe contained only the two E2 binding sites (BS1 and BS2), and EMSAs were similarly performed with titrations of E1 (43 nM to 347 nM) and constant E2 concentration (2.8 nM).

We analyzed the role of putative E1 binding site, if any, in the loading of E1 helicase onto the HPV origin by performing EMSA using radiolabeled DNA probes (described in Supplementary Table S1): one containing the putative E1 binding site plus two E2 binding sites (Figure 6a), and another containing only the two E2 binding sites (Figure 6b). Both DNA probes, when incubated with E1 and E2 proteins, showed the formation of the E1-E2-DNA complex, referred to as the C3* complex. The only difference observed was in the migration patterns of the free probes. The probe with only the two E2 binding sites migrated faster due to its smaller size (50 bp long) compared to the probe containing both the E2 binding sites and the putative E1 binding site (124 bp).

These results demonstrated that the putative E1 binding site was not required for the assembly of the E1-E2-DNA complex at the origin, or that such a binding site does not exist at all, at least based on the results of the EMSA investigation. This finding contrasted with earlier studies, primarily based on the BPV replication initiation model, which suggested that the formation of the E1-E2-DNA complex occurred separately when both proteins bound to their specific sites at the origin [14,40,41,42]. Instead, our data suggested a regulated mechanism in which the putative E1 binding site did not appear to play a role in E1-E2-DNA complex formation. The binding of these two proteins to DNA appeared to be temporal, with E2 binding to its sites on the origin first and then recruiting E1 helicase to the E2-origin complex, directing its binding and thereby possibly initiating DNA melting.

### 2.6. E1-E2-DNA Complex Formation Requires at Least Two E2 Binding Sites

**Figure 7:**
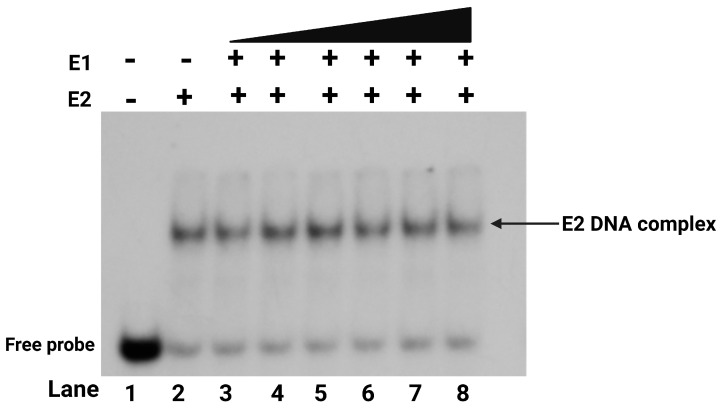
E1-E2-DNA complex formation requires at least two E2 binding sites. Electrophoretic mobility shift assays (EMSAs) were performed to assess E1-E2-DNA complex formation using radiolabeled oligonucleotides containing a single wild-type E2 binding site. Reactions contained a fixed ^32^P-labeled E2 binding site DNA probe (70 pmol), a constant E2 concentration (2.8 nM), and varying E1 protein concentrations (43 nM, 87 nM, 173 nM, and 347 nM).

We further studied the role of multiple E2 binding sites, common in all HPV origins, in E1-E2-DNA ternary complex formation by performing EMSA with a DNA probe (Oligo III, as mentioned in Supplementary Table S1) containing only a single E2 binding site (Figure 7). The results showed that the presence of only one E2 binding site did not support the formation of the E1-E2-DNA complex even in the presence of E2. When E2 was incubated with the probe, the typical E2 dimer-bound C1* complex was observed. However, the addition of E1 did not result in the formation of the higher-order C2* or C3* E1-E2-DNA complex. Even when increasing amounts of E1 protein were titrated, the E1-E2-DNA complex was not observed with DNA sequences containing a single E2 binding site. In contrast, EMSA results with the DNA probe containing two E2 binding sites, as shown in Figure 6b, demonstrated the formation of the E1-E2-DNA (C3*) complex even at a low E1 concentration. These findings indicated that the presence of only one E2 binding site may not be sufficient for loading E1 into the complex formation, emphasizing the requirement for at least two E2 sites to facilitate the formation of the E1-E2-DNA ternary complex.

## 3. Discussion

HPV E1 and E2 are essential HPV-encoded proteins that initiate and regulate DNA replication at the viral origin. E2 functions as the initiator protein by binding to specific DNA sequences at the origin to activate the replication process. E1, as a DNA helicase, unwinds the DNA, creating a replication fork to facilitate viral genome replication. Similar to other dsDNA viruses such as SV40 and Polyomavirus, the HPV family extensively relies on the host’s cellular DNA replication machinery for essential replication factors, including DNA polymerases, topoisomerase I, replication protein A, etc. [48,49,50]. Therefore, understanding the biochemical properties and interactions of these viral proteins is essential to elucidating the mechanism of HPV DNA replication initiation. The prevailing model for papillomavirus replication initiation has been largely derived from studies of bovine virus, and despite some conservation between bovine and human papillomaviruses, significant differences in the viral physiology of BPV and certain HPVs underscore the necessity of studying HPV replication models. Our study offers comprehensive biochemical characterization of the E1 helicase protein and examines the formation and properties of the E1-E2-DNA pre-initiation complex at the HPV origin.

### 3.1. Purification and Functional Characterization of Full-Length HPV16 E1 Helicase

Recombinant full-length HPV16 E1 helicase was purified from *E. coli* for comprehensive biochemical characterization. The oligomeric state of the purified E1 protein was determined using dynamic light scattering, which confirmed that E1 exists as a hexamer in solution (Figure S3 in Supplementary Materials). These findings are in agreement with previous studies showing that HPV11 E1 also forms a hexamer in solution [51,52].

Purified full-length HPV E1 protein exhibited intrinsic ATPase activity (Figure 2 and Figure 3). Kinetic analysis showed that E1 hydrolyzed ATP with a *V_max_* of 37 ± 0.4 nmol/min/mg and a *K_m_* of 118 ± 19 μM for ATP, without ssDNA. In contrast, the M13mp19 ssDNA increased E1’s *V_max_* to 78 ± 0.4 nmol/min/mg, and the *K_m_* to 208 ± 13 μM. This increase in the *V_max_* and *K_m_* indicated that ATP hydrolysis by E1 was facilitated by its binding to long ssDNA. The observed increase in *K_m_* for ATP in the presence of ssDNA suggests a reduced affinity for ATP. However, the increased *V_max_* likely indicates a higher turnover rate, likely due to enhanced catalytic efficiency resulting from facilitated enzyme-substrate interactions with ssDNA. This suggests that while ATP binding is somewhat reduced, it may have helped increase the ATP turnover, and the overall reaction rate increases due to enhanced enzyme activity. The kinetic parameters of E1 are in agreement with previous reports on the E1 protein from different papillomaviruses on its ATPase activity [53]. Previous studies on BPV1 E1 have shown a *K_m_* of 200 µM for ATP hydrolysis [26], while HPV11 E1 has a reported *K_m_* of 380 µM [52] and HPV6b E1 co-purified with E2 has a *K_m_* of 230 µM [35]. We have examined the helicase activity of E1 protein using a fluorescent partial duplex DNA substrate containing a 50 bp double-stranded region with 5 bp 5′ and 3′ forks. The results demonstrated concentration-dependent DNA unwinding by E1 helicase (Figure 4). These findings confirmed the helicase activity of the purified full-length E1 helicase and were in agreement with the helicase activity of E1 protein from BPV [54].

### 3.2. E1 ATPase Activity Is Stimulated by M13mp19 ssDNA

We investigated the stimulation of E1 ATPase activity by various DNA substrates. Our findings revealed that E1 ATPase activity showed no significant stimulation by either 40 bp dsDNA or 30 bp ssDNA. In contrast, the 7.25 kb long M13mp19 ssDNA stimulated the E1 ATPase activity, with an ~80% increase above the basal ATPase level. The observed increase in ATPase activity with M13mp19 ssDNA suggested that the longer ssDNA length likely allowed E1 to translocate along the substrate, thereby stimulating the ATPase activity. This highlighted E1’s preference for binding to long ssDNA over shorter ssDNA or dsDNA, as indicated by the minimal stimulated response with dsDNA or oligo dT_30_. Such stimulation of E1 ATPase activity by longer ssDNA substrates is consistent with known behaviors of DNA helicases. Previous studies on the DnaB helicase from *Bacillus anthracis* also reported greater stimulation with M13mp19 DNA compared to other ssDNA substrates [26,53,55]. Furthermore, our results are also in agreement with studies involving the SV40 T-antigen helicase, which shares significant structural homology with HPV E1 helicase and exhibits intrinsic ATPase activity independent of DNA effectors [56,57].

### 3.3. E2 Protein Mediates E1 Binding to the Viral Origin

E1 and E2 play critical roles in the initiation of HPV DNA replication. Our studies, shown in Figure 5a, demonstrated that E1 helicase alone did not bind to the putative E1 binding site on the origin DNA. Instead, the E1 helicase required the presence of E2 bound to its specific binding sites, which facilitated the recruitment of E1 to form the E1-E2-DNA ternary complex. This interaction is critical, as it highlights the essential role of E2 in recruiting E1 helicase to the viral origin or the initiation complex, a necessary step for initiating DNA replication. E1 appeared to form a di-hexameric complex with E2 and DNA, upon binding, resulting in a larger complex at the origin when sufficient E1 and E2 proteins were present (Figure 5b). These results emphasized that E1’s binding to the origin was possibly indirect, likely mediated through its interaction with E2, which is known to bind specifically to its cognate binding sites. Thus, the E2-DNA complex loads the E1 DNA helicase to the origin, forming the ternary complex. The E1 binding site was originally proposed in BPV, where six thymidine-rich E1 binding sites and an adjacent AT-rich region were shown to facilitate E1 loading [58]. Based on sequence similarity, similar T-rich E1 binding sites were also annotated as E1 binding sites in the HPV genome. However, none of these binding sites appear to be sequence specific. The E1 protein is a DNA helicase and not an origin-binding protein; the origin DNA recognition and binding are carried out by the E2 protein. Consequently, E1 binds to all DNA sequences but nonspecifically, as observed in previous studies and our current investigation.

### 3.4. E1 Binding Site Is Dispensable for E1-E2-DNA Complex Formation

The results presented in Figure 6 demonstrated that the putative E1 binding site did not appear to play any role for E1 to bind to the origin DNA and did not enhance the formation of the E1-E2-DNA complex. The data revealed that E1 is associated with the E2-DNA complex even without the E1 binding site. This indicated that the recruitment of E1 to the viral origin primarily depended on its interaction with E2, which binds to its cognate sites on the DNA. Thus, E2 acted as a regulator for E1 origin binding, facilitating E1’s localization to the origin. While the putative E1 binding site did not contribute to E1 binding, the AT-rich sequence in that region may serve as the initial site for helicase-mediated origin melting. This initiates DNA unwinding, which is a common characteristic among helicases that initiate DNA melting at AT-rich sequences [58,59,60,61].

### 3.5. Two E2 Binding Sites Are Essential for E1-E2-DNA Complex Formation

We assessed the formation of the ternary E1-E2-DNA complex using DNA probes, one with a single E2 binding site and the other with two E2 binding sites. E2 alone formed a single complex with the DNA probe with one binding site (Figure 7); however, titration with E1 did not lead to any higher-order complex, as no additional shifts were detected even at higher E1 concentrations. Our results in Figure 6b using a DNA probe with two E2 binding sites demonstrated that at least two E2 binding sites were essential for assembly of the E1-E2-DNA pre-initiation complex. Alternatively, it is likely that E1 requires two E2 dimers bound at the viral origin to serve as a large platform for E1 binding. This DNA-binding arrangement reflected the cooperative interplay between E1 and E2 in binding the origin. These findings provided a mechanistic explanation for the requirement of multiple E2 binding sites in all HPV origins of DNA replication for the initiation of HPV DNA replication.

**Figure 8:**
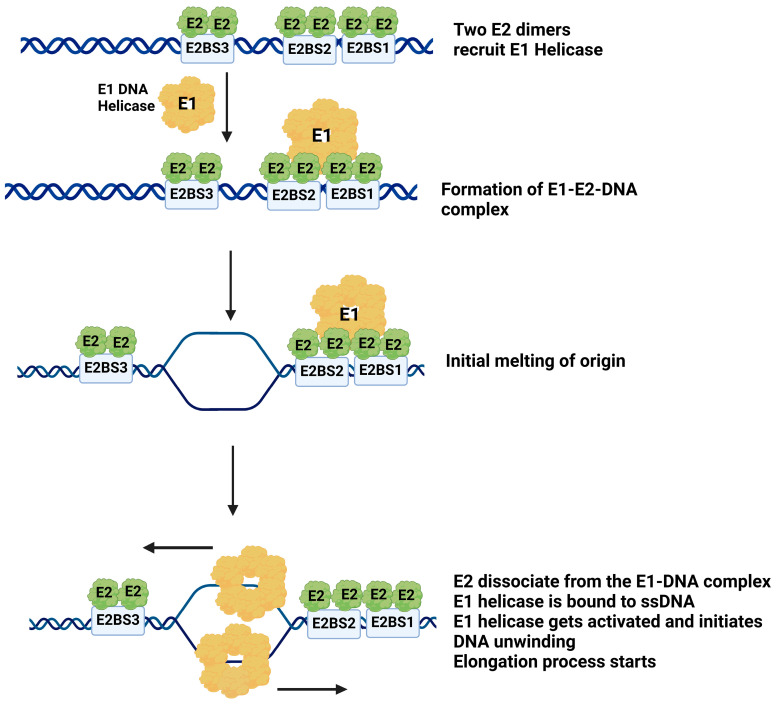
Putative model for E1-E2 complex assembly and HPV DNA replication initiation. A proposed model for the assembly of the E1-E2 complex at the origin to initiate HPV DNA replication is presented. The initial step involves the binding of E2 dimers at two of the E2 binding sites located within the origin. Subsequently, E2 facilitates the recruitment of E1, resulting in the formation of an E1-E2-DNA complex. This complex formation induces localized melting of the DNA, likely exposing single-stranded DNA, and positions E1 for helicase activity initiation. At this stage, E2 may no longer be necessary. The dissociation of E2 from the E1-DNA complex likely marks the next phase of the HPV DNA replication. As per this model, E1 is recruited to the origin through E2 bound to BS1, BS2, and BS3, which are generally conserved across high-risk and low-risk HPV types. Our previous results showed that E2 binds all four of its binding sites in high-risk and low-risk HPV types, with only minor differences in affinity depending on the mutation(s) on the consensus sequence. Since E1 loading is E2-dependent, this mechanism is likely conserved across the HPV genus.

Based on the data presented here, we propose a model (Figure 8) illustrating the interplay between E1 and E2 proteins at the viral origin during the initiation of DNA replication. HPV DNA replication initiates when multiple E2 dimers bind to their specific binding sites at the origin [62]. Our EMSA assay confirmed that at least two E2 binding sites were essential for recruiting the E1 helicase. E1 helicase localizes to the viral DNA origin primarily through interactions with the E2 protein, which is already bound to the DNA. This finding aligns with previous research showing direct interactions between E1 and E2, as demonstrated by the X-ray structure of the HPV18 E2 activation domain bound to the E1 helicase domain [63]. Crucially, the formation of the E1-E2-DNA complex does not strictly require the presence of the E1 binding site on the DNA. Instead, E1 recruitment to the origin is driven by its interactions with E2 protein in the E2-DNA complex, rather than necessitating direct binding of E1 to the putative E1 binding site at the origin DNA. The proximity of the putative E1 binding site to the cluster of E2 binding sites (BS1, BS2, and BS3) suggests that E2 dimers play a critical role in localizing E1 to the origin. The binding of E1 and E2 near the E2 binding sites 1, 2, and 3 may induce torsional strain or partial unwinding of the AT-rich region at the origin. This strain likely facilitates the initial melting of the DNA. As a result, E1 can unwind the origin DNA, establishing the replication fork. After the initial melting of the origin DNA, E2 dissociates from the E1-DNA complex, as ATP acts as an allosteric effector promoting this dissociation. This dissociation is crucial for E1 to assemble into an active helicase and proceed with DNA unwinding [63].

This process in HPV is reminiscent of the *E. coli* replication model. *E. coli* DNA replication, first outlined by Kornberg, serves as a model for understanding replication in various organisms, including HPV, with both systems sharing similarities in initiation mechanisms [64]. *E. coli* DNA replication initiates at the oriC, which contains four binding sites for the initiator protein DnaA. When DnaA binds to these sites, it promotes the unwinding of an AT-rich DNA segment. The helicase loader protein DnaC then aids in loading the DnaB helicase onto the unwound DNA strand. The DnaB complex further separates the DNA strands, allowing single-stranded DNA binding proteins and other replication machinery to assemble and initiate replication [60,65]. Similar to the *E. coli* replication, our proposed model suggests that E1 and E2 binding near the AT-rich region may induce torsional strain, leading to initial DNA melting. However, unlike *E. coli*, HPV replication does not involve any helicase loader protein, which in fact supports our hypothesis that E2 plays a critical role in recruiting the E1 helicase to the viral origin, potentially facilitating the initiation of DNA unwinding and replication, in a regulated manner. It is important to note that this study focuses specifically on the early steps of replication initiation mediated by E1 and E2 proteins. Host factors come into play in the subsequent stages of viral DNA replication. Future studies should explore the recruitment of host factors to the initiation complex using purified cellular factors.

## 4. Materials and Methods

All chemicals, buffers, and reagents used in this study were ACS reagent grade and obtained from Fisher Scientific, Pittsburgh, PA, USA.

### 4.1. Buffers

Buffer A contained 25 mM NaH_2_PO_4_ pH 7.5, 5 mM imidazole, and 500 mM NaCl; Buffer B contained 25 mM NaH_2_PO_4_ (pH 7.5), 85 mM imidazole, and 500 mM NaCl; Buffer C contained 25 mM NaH_2_PO_4_ (pH 7.5), 250 mM imidazole, and 500 mM NaCl; Buffer A_0_ contained 25 mM NaH_2_PO_4_ pH 7.5, 500 mM NaCl, 5 mM MgCl_2_, 10% glycerol, 0.01% NP-40, and 1 mM DTT; Buffer D contained 10 mM Tris pH 7.5 and 100 mM sodium chloride; and 1X TBE contained 89 mM Tris−borate (pH 8.3) and 2.5 mM EDTA.

### 4.2. Expression and Purification of Full-Length HPV E1 and E2 Proteins

The full-length HPV16 E1 and E2 genes were codon-optimized using GeneOptimizer technology and synthesized through GeneArt Gene Synthesis (Thermo Fisher, Regensburg, Germany). Both genes were engineered with a C-terminal 10× histidine tag for purification. The E1 and E2 coding sequences were cloned into the pET28α expression vector and transformed into BL21 (DE3) RIPL cells for protein expression. For E1, protein expression was induced with 0.4 mM IPTG at 18 °C overnight, while E2 expression was induced at 13 °C. After induction, cells were harvested by centrifugation at 4000 rpm, and the pellet was resuspended in lysis buffer (25 mM Tris pH 7.5, 10% sucrose, 5 mM MgCl_2_, 150 mM NaCl). Cells were subjected to one cycle of freeze-thawing and were treated with 0.2 mg/mL lysozyme. Soluble proteins were extracted using the BugBuster reagent (MilliporeSigma Burlington, MA, USA) and 500 mM NaCl. The lysates were diluted in buffer A and loaded onto Ni^2+^-charged His-bind resin (Novagen Inc., Milwaukee, WI, USA) for affinity purification. After extensive washing with buffer B, bound proteins were eluted with buffer C and concentrated by dialysis in buffer A_0_ for 8 h.

### 4.3. ATPase Assay

ATPase assays were carried out according to previously described methods [55,66,67]. A standard 10 µL reaction mixture contained 100 μM ATP-α-^32^P (PerkinElmer, Waltham, MA, USA), different amounts of purified E1 protein (0–0.9 μg), and different ssDNA substrates in buffer (25 mM Tris-HCl (pH 7.5), 10% (*v/v*) glycerol, 0.1 mg/mL BSA, 5 mM MgCl_2_, and 5 mM DTT). Reactions were incubated at 37 °C for 60 min. The reactions were then spotted onto polyethyleneimine-cellulose strips that were pre-spotted with 5 mM ADP/5 mM ATP solution. The strips were developed using 1 M formic acid and 0.5 M LiCl in a thin-layer chromatography chamber and then dried. Parts of the strips containing ADP and ATP were cut separately and counted using a liquid scintillation counter (Perkin Elmer Tri-Carb 2900, Shelton, CT, USA). ATPase activity (pmol/min) was calculated and further analyzed using GraphPad Prism 5.0. The ATPase assays were performed in three independent replicates, with error bars representing the standard deviations.

### 4.4. Kinetic Analysis of E1 ATPase Activity

To measure the kinetic parameters of E1 ATPase activity, a standard ATP hydrolysis assay was conducted with titrations of ^32^P-ATP ranging from 0 to 0.75 mM. The reaction mixture contained 240 ng of E1 protein and 100 pmol of M13mp19 ssDNA, unless otherwise specified. The remaining assay was performed as the standard assay. Data collected from the assay were analyzed using GraphPad Prism 5.0, which was used to calculate the *K_m_* and *V_max_* values for the E1 protein. The kinetic assay was performed in three independent replicates, with error bars representing the standard deviations.

### 4.5. DNA Helicase Assay

The helicase assays were conducted as previously described [66,68,69,70,71], with a few modifications. The DNA substrate used in the assay was prepared by annealing a synthetic 60-mer oligonucleotide with the M13mp18 ssDNA. The oligonucleotide was complementary to a 50 bp region between nucleotides 6268 and 6317 of M13mp18 ssDNA and contained five-nucleotide nonhomologous tails at both the 5′ and 3′ ends. In addition to this, the 60-mer oligonucleotide also contained a FAM fluorescent tag at the 5′ end. After annealing, the partially duplex DNA substrate was purified in buffer D using a Sephacryl S-400 column (Cytiva, Marlborough, MA, USA).

The purified FAM-labeled DNA substrate was used for the E1 helicase assay, in which a standard 10 µL reaction mixture contained 5 mM MgCl_2_, 3.5 mM ATP, labeled DNA substrate, and the specified amount of E1 protein in buffer (25 mM Tris-HCl (pH 7.5), 10% (*v/v*) glycerol, 0.1 mg/mL BSA, and 5 mM DTT). The mixtures were incubated at 37 °C for 2 h, after which the reactions were terminated by adding 4 μL of a solution containing 2.5% SDS, 60 mM EDTA, and 1% bromophenol blue. These reaction mixtures were then run on 1% agarose gels in a running buffer, which contained 1× TBE and 0.1% SDS. Post-electrophoresis, the gels were imaged using an iBright FL1500 Imaging System (Thermo Fisher Scientific, Waltham, MA, USA), with appropriate fluorescent channels. The imaged bands on the gel were exposed differentially, with the top and bottom bands exposed for varying time periods. Helicase activity was quantitated by analyzing the bottom bands relative to the boiled control substrate, which was included in each of the triplicate assays performed, using iBright analysis software 4.0. The assay was performed in three independent replicates, with error bars representing the standard deviations.

### 4.6. Radiolabeling of Oligonucleotides

Synthetic oligonucleotides of varying lengths were PCR amplified from the HPV-11 upstream regulatory region (URR) and used in mobility shift assays. These oligonucleotides (as mentioned in Supplementary Table S1) were 5′ end-labeled using (γ-^32^P) ATP and T4 polynucleotide kinase (New England Biolabs, Burlington, MA, USA), according to the manufacturer’s protocol. Following labeling, the DNA probes were purified using a Bio-Gel P-6 column (Bio-Rad, Hercules, CA, USA).

### 4.7. Electrophoretic Mobility Shift Assay (EMSA)

The binding reactions were performed in a final volume of 10 µL, containing specified quantities of 11-E1 and 11-E2 proteins and 70 pmol of ^32^P-labeled DNA probe. The reaction buffer comprised 10 mM HEPES (pH 7.9), 4 mM MgCl_2_, 10% glycerol, 5 mM dithiothreitol, 0.2 mM EDTA, 0.1 mg/mL bovine serum albumin, 1.4 mM ATP, and 50 mM NaCl. The reactions were incubated for 10 min at 30 °C instead of 37 °C to minimize potential interference from trace amounts of nucleases, DNases, or proteases. Following the binding reaction, the mixtures were immediately subjected to electrophoresis on a 5% polyacrylamide gel in 0.5× TBE buffer, running at 170 V for 1 h and 15 min at 4 °C. After electrophoresis, the gels were dried and exposed to X-ray films for imaging. Each EMSA was performed in three independent replicates. Densitometric quantitation of C1*, C2*, and C3* complex formation was performed using Image J from EMSA gels generated with increasing E1 concentrations in the presence of E2 and a radiolabeled dsDNA probe. The quantification results are shown in Supplementary Materials (Figure S1).

## 5. Conclusions

This study provides novel insights into the mechanistic role of E2 in the recruitment and activation of the E1 helicase for HPV DNA replication. By investigating the interaction between E1 and E2 at the HPV origin, we reveal that E2 binding to the origin DNA is essential for E1 loading, without the apparent evidence of a specific E1 binding site, providing a model for the HPV replication initiation. These findings improve our understanding of HPV biology and could help in future therapeutic strategies targeting viral DNA replication, including the development of small molecules that disrupt the E1-E2 interaction, to inhibit HPV DNA replication. Future studies should focus on identifying specific residues involved in E1-E2 binding and performing DNA-binding experiments with E1 and E2 mutants that reduce their interaction affinity, which would further elucidate the formation and characterization of the E1-E2-DNA complex.

## Data Availability

All relevant data analyzed during this study are included in the article and its Supplementary Materials. Additional data supporting the findings of this study are available from the corresponding author upon reasonable request.

## Supplementary Materials

The following supporting information can be downloaded at: https://www.mdpi.com/article/10.3390/ijms26094333/s1.

## Abbreviations

The following abbreviations are used in this manuscript:

HPV	Human papillomavirus
BPV	Bovine papillomavirus
EMSA	Electrophoretic mobility shift assay
LCR	Long control region
BS	Binding site
SNV	Single-nucleotide variation
