# Supplementary material for: Mechanisms of Viral DNA Replication of Human Papillomavirus: E2 Protein-Dependent Recruitment of E1 DNA Helicase to the Origin of DNA Replication
Source: Int J Mol Sci. 2025 May 2;26(9):4333. doi: 10.3390/ijms26094333 (PMC12072466; doi:10.3390/ijms26094333)
Supplement: Supplementary file 1 [file ijms-26-04333-s001.zip › ijms-3580800-supplementary.pdf]

Mechanisms of Viral DNA Replication of Human Papillomavirus: E2 Protein-Dependent Recruitment of E1 DNA Helicase to the Origin of DNA Replication

Anshul Rana [1,2], Gulden Yilmaz [3], Esther E. Biswas-Fiss [1,2] and Subhasis B. Biswas [1,*]

[1] Department of Medical and Molecular Sciences, College of Health Sciences, University of Delaware, Newark, DE 19716, USA
[2] Ammon Pinizzotto Biopharmaceutical Innovation Center, Newark, DE 19713, USA
[3] Department of Molecular Biology, Rowan University, Stratford, NJ 08084, USA

List of the materials:

Table S1. Sequences of DNA probes utilized in electrophoretic mobility shift assays.

Figure S1. Densitometric quantification of C1*, C2*, and C3* complex formation with increasing E1 concentration.

Figure S2. Statistical analysis of E1 ATPase activity in the presence of different DNA substrates.

Figure S3. Dynamic light scattering (DLS) analysis of E1 protein.

Oligonucleotide	Sequence
**Oligo I** E2 BS1+2+E1BS (124 bp)	5′GTAACCCACACCCTACATATTTCCTTCTTAT*ACTTAATAACAATCTTAG*TTTAAAAAAGAG-GAGGG**ACCGAAAACGGT**TCA**ACCGAAAACGGT**TATATATAAACCAGCCCAAAAAATTAGCAGA3′
**Oligo II** E2 BS1+2 (50 bp)	5′GGCGGG**ACCGAAAACGGT**TCA**ACCGAAAACGGT**TATATATAAACCAGCCC3′
**Oligo III** E2 BS (24 bp)	5′CTTGCA**ACCGTTTTCGGT**TGCCCT3′

Table S1. Sequences of DNA probes utilized in electrophoretic mobility shift assays. Sequences of only one strand of each duplex oligonucleotide are shown. E2 binding sites are bold and underlined, while the putative E1 binding site is in italics and underlined. The sizes of each oligonucleotide are presented.

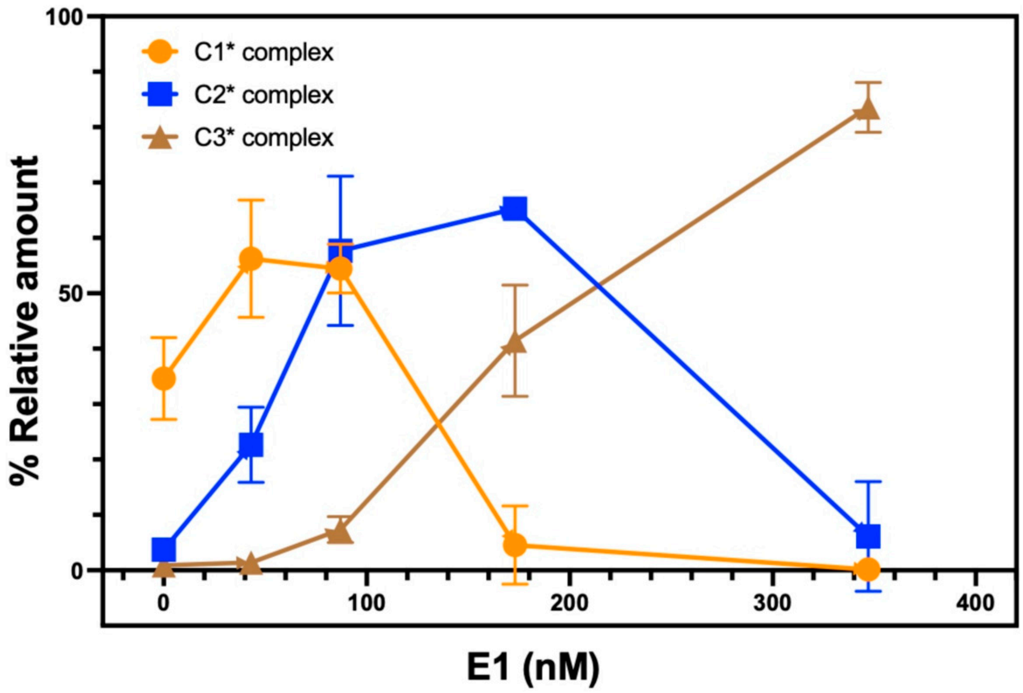

Figure S1. Densitometric quantification of C1*, C2*, and C3* complex formation with increasing E1 concentration. EMSAs were performed using increasing concentrations of E1 (0–347 nM) in the presence of E2 (2.8 nM) and radiolabelled dsDNA probe (oligo I and II). Band intensities corresponding to C1* (●, orange), C2* (■, blue), and C3* (▲, brown) complexes were quantified using ImageJ and plotted as percent relative amount of total shifted DNA. Error bars represent standard deviation from three independent experiments.

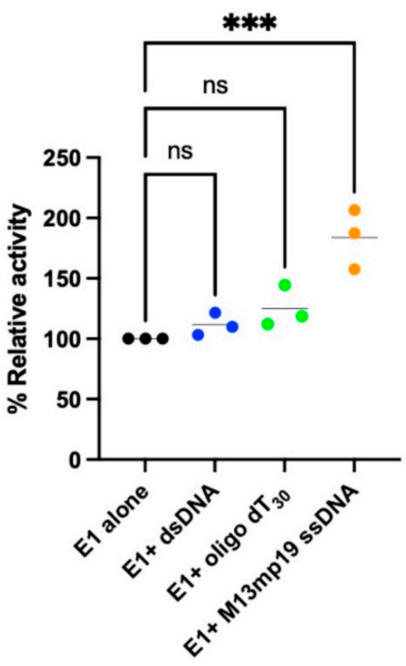

Figure S2. Statistical analysis of E1 ATPase activity in the presence of different DNA substrates. ATPase activity was measured in the presence of dsDNA, oligo dT_{30}, or M13mp19 ssDNA and compared to the no-DNA control. A one-way ANOVA followed by Dunnett's multiple comparisons test was performed using GraphPad Prism 10. M13mp19 ssDNA significantly stimulated E1 ATPase activity (p = 0.0005), while dsDNA (p = 0.7070) and oligo dT_{30} (p = 0.1947) did not show significant differences compared to the control. Data are presented as scatter dot plots with mean ± SD; asterisks indicate statistically significant differences. The corresponding ATPase activity data are shown in Figure 3. *** $p \leq 0.001$.

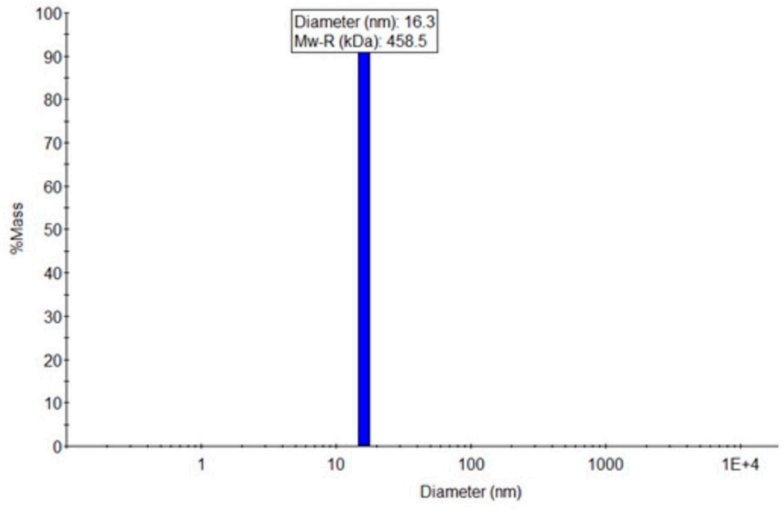

Figure S3. Dynamic light scattering (DLS) analysis of E1 protein. A representative DLS histogram of E1 showed a mass of 458 kDa, which is close to the expected molecular weight of 450 kDa. The molar mass, estimated at 458 ± 6 kDa from a measured diameter of 16.3 ± 0.05 nm, suggests that E1 exists as a hexamer.
